# Weekend specialist intensity and admission mortality in acute hospital trusts in England: a cross-sectional study

**DOI:** 10.1016/S0140-6736(16)30442-1

**Published:** 2016-07-09

**Authors:** Cassie Aldridge, Julian Bion, Amunpreet Boyal, Yen-Fu Chen, Mike Clancy, Tim Evans, Alan Girling, Joanne Lord, Russell Mannion, Peter Rees, Chris Roseveare, Gavin Rudge, Jianxia Sun, Carolyn Tarrant, Mark Temple, Sam Watson, Richard Lilford, HiSLAC Collaborative

**Affiliations:** a University of Birmingham, Birmingham, UK; b University Hospitals Birmingham NHS Foundation Trust, Birmingham, UK; c University of Warwick, Coventry, UK; d University Hospitals Southampton NHS Foundation Trust, Southampton, UK; e Royal Brompton & Harefield NHS Foundation Trust, London, UK; f University of Southampton, Southampton, UK; g Academy of Medical Royal Colleges Patient Liaison Group, London, UK; h Southern Health NHS Foundation Trust, Southampton, UK; i University of Leicester, Leicester, UK; j Heart of England NHS Foundation Trust, Birmingham, UK

## Abstract

**Background:**

Increased mortality rates associated with weekend hospital admission (the so-called weekend effect) have been attributed to suboptimum staffing levels of specialist consultants. However, evidence for a causal association is elusive, and the magnitude of the weekend specialist deficit remains unquantified. This uncertainty could hamper efforts by national health systems to introduce 7 day health services. We aimed to examine preliminary associations between specialist intensity and weekend admission mortality across the English National Health Service.

**Methods:**

Eligible hospital trusts were those in England receiving unselected emergency admissions. On Sunday June 15 and Wednesday June 18, 2014, we undertook a point prevalence survey of hospital specialists (consultants) to obtain data relating to the care of patients admitted as emergencies. We defined specialist intensity at each trust as the self-reported estimated number of specialist hours per ten emergency admissions between 0800 h and 2000 h on Sunday and Wednesday. With use of data for all adult emergency admissions for financial year 2013–14, we compared weekend to weekday admission risk of mortality with the Sunday to Wednesday specialist intensity ratio within each trust. We stratified trusts by size quintile.

**Findings:**

127 of 141 eligible acute hospital trusts agreed to participate; 115 (91%) trusts contributed data to the point prevalence survey. Of 34 350 clinicians surveyed, 15 537 (45%) responded. Substantially fewer specialists were present providing care to emergency admissions on Sunday (1667 [11%]) than on Wednesday (6105 [42%]). Specialists present on Sunday spent 40% more time caring for emergency patients than did those present on Wednesday (mean 5·74 h [SD 3·39] *vs* 3·97 h [3·31]); however, the median specialist intensity on Sunday was only 48% (IQR 40–58) of that on Wednesday. The Sunday to Wednesday intensity ratio was less than 0·7 in 104 (90%) of the contributing trusts. Mortality risk among patients admitted at weekends was higher than among those admitted on weekdays (adjusted odds ratio 1·10, 95% CI 1·08–1·11; p<0·0001). There was no significant association between Sunday to Wednesday specialist intensity ratios and weekend to weekday mortality ratios (*r* −0·042; p=0·654).

**Interpretation:**

This cross-sectional analysis did not detect a correlation between weekend staffing of hospital specialists and mortality risk for emergency admissions. Further investigation is needed to evaluate whole-system secular change during the implementation of 7 day services. Policy makers should exercise caution before attributing the weekend effect mainly to differences in specialist staffing.

**Funding:**

National Institute for Health Research Health Services and Delivery Research Programme.

## Introduction

Provision of 7 day health services is a key policy for the UK Government,^1^ a strategic objective for the English National Health Service (NHS),^2^ and a topic of potential interest to other health systems trying to maximise efficient use of infrastructure and resources.^3^ Policy makers have focused particularly on the increased mortality associated with weekend admission (the so-called weekend effect), first described in 2001, by Bell and Redelmeier,^4^ who suggested that this effect might be attributable to reduced hospital physician staffing at weekends. Since then, the weekend effect has been shown to be ubiquitous in all high-income health systems in which it has been studied;^5^ the relative risk increase for unselected emergency admissions in England and the USA is estimated to be 16%,^6^ translating into many thousands of lives lost each year.

Both the UK Secretary of State for Health^7^ and the Department of Health^8^ have explicitly attributed the weekend effect to reduced availability of hospital doctors, particularly consultants, stating that changes to doctors' employment contracts will be needed. In 2013, NHS England published ten standards for 7 day services, relating to emergency admissions at weekends, to be implemented by 2016–17,^9^ of which six standards specifically require that care should be directed or delivered by consultants or senior decision makers.

Despite professional and public support for improved senior medical staffing of hospitals at weekends,^9^, ^10^ evidence that this approach will reduce the weekend effect is elusive. Alternative explanations for the weekend effect include selection bias through casemix variation,^11^, ^12^ restricted access to diagnostic services,^13^ and (for stroke) reduced nurse staffing.^14^ Potential mechanisms by which specialists might mitigate the weekend effect include reduced error and improved diagnostic and therapeutic accuracy.^14^, ^15^, ^16^, ^17^ Although physician intensity has been associated with worse outcomes at the organisational level,^18^, ^19^, ^20^ it has not been associated specifically with the weekend effect.^14^ The scarcity of evidence for physician staffing is problematic because the potential costs of implementation of 7 day services (including increased consultant presence at weekends) are estimated at £1·07 billion to £1·43 billion.^21^

**Research in context**

**Evidence before this study**

Increased availability of hospital specialists (consultants) at weekends is central to the provision of 7 day services and has been proposed as the solution to the so-called weekend effect—the increased mortality associated with weekend admission to hospital reported by many health systems worldwide. Policy makers need to identify whether increased presence of specialists at weekends is a cost-effective intervention. The HiSLAC project addresses this need. We searched MEDLINE, CINHAL, Embase, HMIC, EThOS, and the Cochrane Library from Jan 1, 2000, to April 15, 2015, for quantitative and qualitative publications relating to the weekend effect, with no language restrictions. Our search terms were “weekend/weekday” or “out-of-hours”, and “hospital admissions”. Our search retrieved 5404 publications, of which 959 were considered potentially relevant. No report quantified the difference in specialist staffing between weekends and weekdays and explicitly linked the deficit in specialist staffing to the magnitude of the weekend effect across specialties at national level. Weekends are associated with higher error rates than weekdays, and with more failure-to-rescue events linked to reduced nursing intensity. The weekend effect might also be attributable to increased severity of illness of patients admitted at weekends.

**Added value of this study**

To our knowledge, this is the first study in a national health system to quantify specialist involvement in the care of emergency admissions at weekends and weekdays, and to analyse this with weekend and weekday admission mortality rates. We found no correlation across different trusts between the mortality risk for emergency admissions at weekends and the relative levels of specialist involvement on Sundays and Wednesdays.

**Implications of all the available evidence**

The two-phase HiSLAC project will evaluate the whole-system secular change during the implementation of 7 day services from 2014 to 2018. At this stage, policy makers should be cautious in ascribing the weekend effect to a single component in a complex system.

With the High-intensity Specialist Led Acute Care (HiSLAC) project, we aim to test the hypothesis that the weekend effect is attributable to reduced senior staffing, using the natural experiment offered by the roll-out of NHS England's 7 day services in acute hospital trusts in England over 5 years (2014–18), which includes enhanced medical staffing at weekends. The study is done in two phases. The first phase is reported here and examines preliminary associations between intensity and weekend admission mortality across the NHS. Phase 2 will take place over the next 4 years to evaluate secular changes in these variables.

## Methods

### Study design and procedures

Eligible participant trusts were those in England receiving unselected emergency admissions. Each trust was invited to appoint a HiSLAC local project lead. Endorsement for the project was obtained from NHS England, the Academy of Medical Royal Colleges and patient representatives, and the NHS Confederation. The National Research Ethics Committee approved this phase of the project as service evaluation of an existing form of health-care delivery without collecting patient-identifiable data.

We convened a moderated workshop of delegates with expertise in hospital medicine, human resources, and health services research, with follow-up by email. We defined specialists as consultants or associate specialists with a certificate of completion of specialist training or equivalent. Two instruments were developed and piloted before implementation: a point prevalence survey designed to acquire information as close to the bedside as possible, and a directorate-level managerial questionnaire (appendix).

The link to the web-enabled point prevalence survey was distributed by email to all specialists in participating trusts by local project leads by use of trust-generated distribution lists. The survey sought non-attributable data relating to the care of emergency admissions on 2 days in June, 2014: Sunday 15th and Wednesday 18th. Sunday and Wednesday were selected because they are associated with the highest and lowest admission mortality risks, respectively,^6^ and specialist intensity is likely to be at its lowest on Sunday. June was chosen because it contains no public holidays in England and is unaffected by winter pressures. Respondents were asked to confirm specialist status, their specialty and location in the hospital, and to estimate the number of hours they had spent between 0800 h and 2000 h on each of these days specifically caring for patients who had been admitted as emergencies. Local project leads were emailed four reminders in the month following these dates.

The managerial directorate-level questionnaire focused on four acute specialties central to the emergency non-operative patient pathway: the emergency department (including the clinical decision unit), acute medical unit, intensive care unit, and acute medical wards receiving acute medical emergencies. The questionnaire was distributed 4 months after the point prevalence survey by local project leads to the clinical directors of these services. The aims of the questionnaire were to cross-validate information about specialty intensity with the point prevalence survey, and to gain additional information about specialist practice in relation to 7 day services.

### Statistical analysis

Specialist intensity at each trust is defined as the self-reported estimated number of specialist hours per ten emergency admissions between 0800 h and 2000 h on Sunday and Wednesday. The emergency admission rate was obtained from the Hospital Episode Statistics dataset and calculated as the mean for that day (Sunday or Wednesday, 24 h) throughout the financial year 2013–14. The imperfect correspondence between time windows for numerator and denominator takes into account the fact that specialists would have been attending emergency patients who had been admitted throughout the entire 24 h, not just those admitted during the day, and the absence of a time stamp in Hospital Episode Statistics data. Because not all specialists responded to the questionnaire, and in view of variable response rates between trusts, estimates of total specialist hours from the point prevalence survey were scaled up with the reciprocals of the survey response rates in each trust.

Directorate-level questionnaire data for the four acute specialties were correlated with the corresponding trust data from the point prevalence survey to test data validity. We used the Sunday to Wednesday intensity ratio to quantify the relative levels of specialist engagement on those days. Scaling for response rates had no effect on Sunday to Wednesday ratios. To account for trust size, we stratified trusts by quintiles, with bed numbers acquired from the mean average of NHS England's (KH03) quarterly submissions for 2014,^22^ or with data from the most recent Care Quality Commission inspection.^23^

We obtained data for emergency hospital admissions for financial year 2013–14, from the Health and Social Care Information Centre. We analysed in-hospital mortality using a logistic regression model at the individual patient level for adult patients undergoing emergency admission, excluding patients younger than 16 years and primary maternity admissions. We included interaction terms for trust, day of admission (Monday to Sunday), sex, age (using a restricted cubic spline with 5 knots), the income deprivation component of the Index of Multiple Deprivation 2010, diagnostic category (as represented by the Clinical Classification Software code, and a categorised index of comorbidity. The comorbidity term includes the three categories of the Charlson score used by the English NHS Summary Hospital-Level Mortality Indicator.^24^ This approach is similar to that of Freemantle and colleagues.^6^ We used this model to obtain adjusted estimates of system-wide admission-day and weekend effects. A system-wide estimate of the weekend effect was calculated as the difference (on a log odds scale) between the average of the Saturday and Sunday coefficients and the average of the five weekday coefficients.

To investigate the effect of weekend admission in each trust, the model was refitted with additional trust × weekend terms to indicate admission by each trust on a Saturday or Sunday. The coefficients of these terms were added to the system-wide weekend effect estimate in the refitted model to obtain trust-level weekend effects. These effects are reported as trust-specific odds ratios (ORs). We investigated the association between the trust-specific weekend ORs and the specialist intensity ratios using correlation methods. The focus on ratios within trusts, rather than absolute levels of weekend mortality and specialist intensity, minimises the effect of unmeasured differences in casemix and other potential trust-level confounders.

Data extraction was done with Microsoft SQL server 2008. We fitted models with Stata (version 14.1).

### Role of the funding source

The funders of the study had no role in the study design, data collection, data analysis, data interpretation, or writing of the report. The corresponding author had full access to all the data in the study and had final responsibility for the decision to submit for publication.

## Results

Of 141 eligible acute hospital trusts, 127 trusts agreed to participate and appointed a local project lead. The appendix shows the geographical distribution of participating trusts. 96 (76%) trusts had one site with an emergency department, 26 (20%) trusts had two sites, and five (4%) trusts had three sites. 115 (91%) of the 127 participating trusts contributed data to the point prevalence survey. Of 34 350 clinicians surveyed, 15 537 (45%) responded (table 1), with response rates ranging from 45 (16%) of 276 responders to 309 (79%) of 393 responders, and exceeding 40% in two-thirds of trusts. 1003 (6%) responders did not hold a specialist accreditation and two (<1%) had incomplete responses and were excluded from further analysis (table 1).

Substantially fewer specialists were present and providing care to emergency admissions on Sunday than on Wednesday (table 1). This difference was partly offset by the greater average time (40%) spent in the care of acutely admitted patients per specialist present on Sunday than on Wednesday (table 1). These patterns were consistent across the quintiles of trust size (table 1).

**Figure 1:**
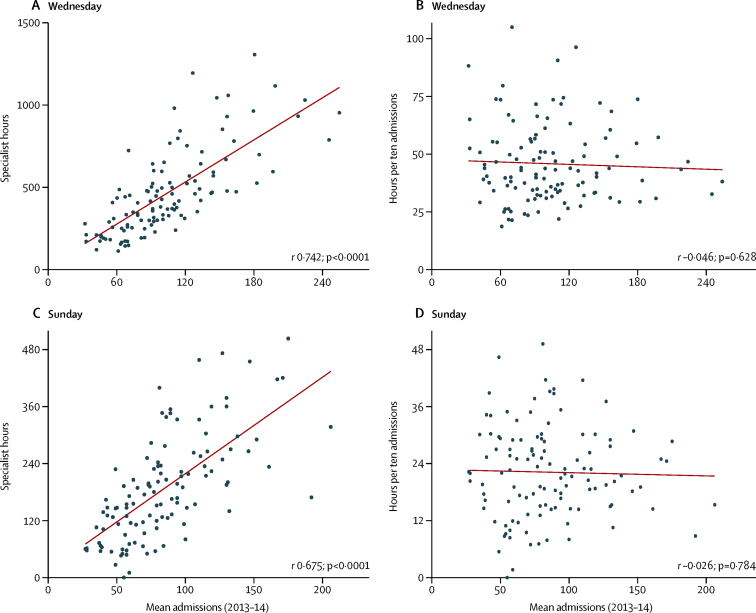
Specialist hours, specialist intensity, and emergency admissions The figure shows the estimated total hours for specialists attending emergency admissions on Wednesday, June 18, 2014 (A), and Sunday, June 15, 2014 (C), against the mean number of emergency admissions for Wednesdays or Sundays in 2013–14, for the 115 trusts responding to the point prevalence survey; and the specialist intensity measure (hours per ten emergency admissions) against the mean number of admissions for Wednesdays or Sundays (B, D). Pearson correlations (*r*) and p values are shown.

For both Sunday and Wednesday, a clear association was evident between the sum of specialist hours delivered by each trust and the numbers of emergency admissions on that day averaged across the year: larger hospitals had more specialists and more admissions (figure 1). There was substantial variation between trusts in the estimated number of hours delivered for the same mean admission rate (figure 1). However, the association with emergency admissions disappeared when specialist hours were expressed per ten emergency admissions (figure 1), with wide variation particularly among the medium to smaller trusts in terms of specialist intensity at any given emergency admission volume.

**Figure 2:**
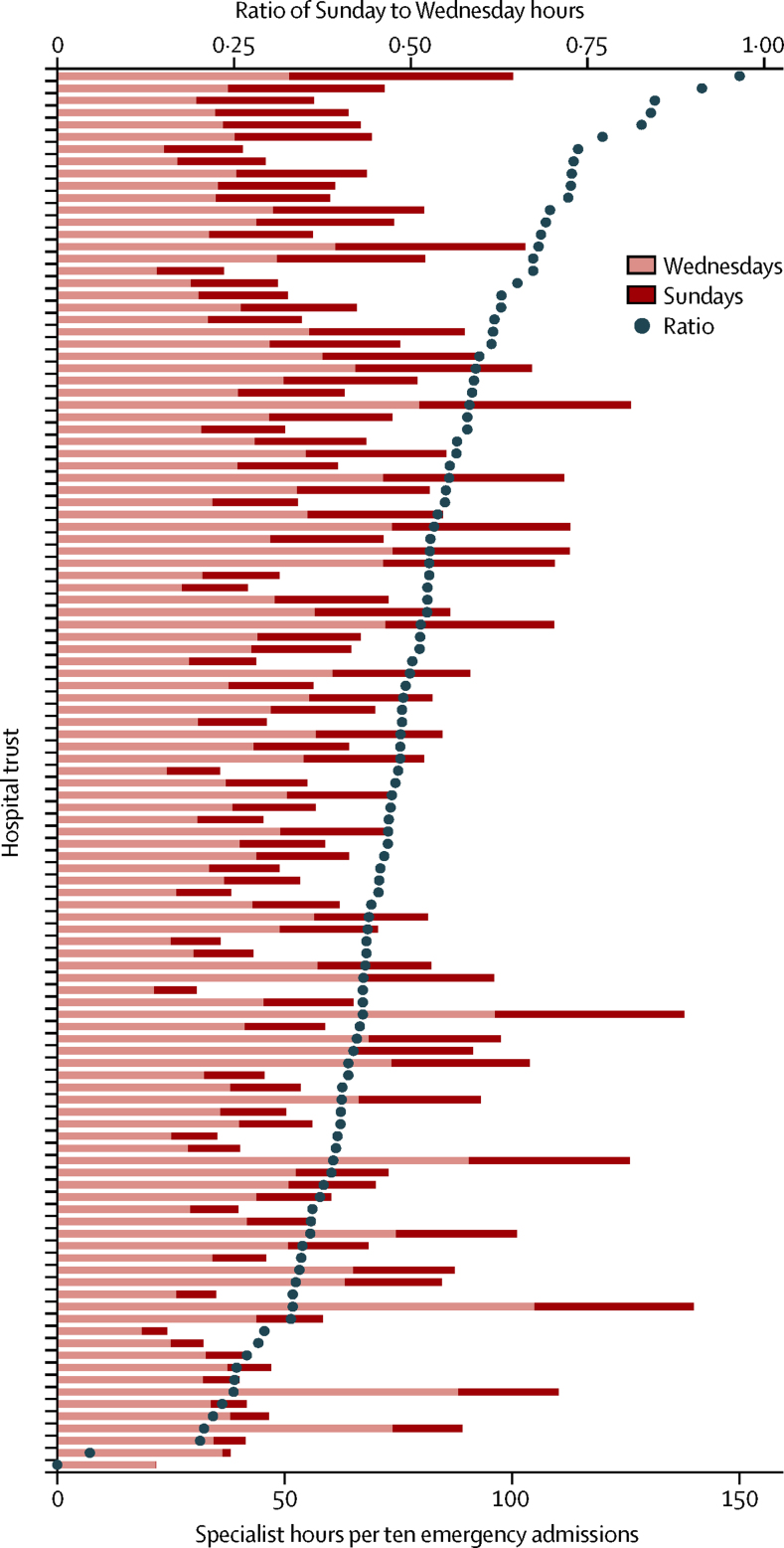
Specialist intensity by trust For each trust, the bars represent specialist hours per ten emergency admissions from the point prevalence survey for Wednesday, June 18, 2014, and Sunday, June 15, 2014. Trusts are shown in decreasing order of the plotted intensity ratios, defined as the relative sizes of the bars.

Factors other than emergency workload might affect the amount of specialist time delivered to emergency admissions in any given trust. To adjust for workload, specialist intensity estimates are expressed in relation to the number of emergency admissions in table 1 and figure 2. The median Sunday to Wednesday intensity ratio was 0·48 across all trusts, with similar results within each quintile of trust size (table 1). No trust had a ratio that was greater than 1, and in 104 (90%) of the 115 contributing trusts the ratio was less than 0·7 (figure 2).

These results are necessarily reliant on the 45% of specialists who responded to the survey. To investigate the possibility that specialists who were present on the survey days were more likely to respond than those who were not, we examined the correlations between the trust response rates and the proportions of specialists present on Wednesday (*r* −0·023; p=0·810) and on Sunday (−0·184; p=0·049). These correlations were fairly small, suggesting that any responder bias present most likely occurs in a similar manner across all trusts, although its presence cannot be completely discounted.

For the directorate-level questionnaire, 40 (31%) of the 127 participant trusts provided complete responses for all four clinical service areas involved in the care of non-operative emergency admissions (emergency department [plus clinical decision unit], acute medical unit, intensive care unit, and acute medical wards receiving acute medical emergencies); 41 (32%) trusts supplied partial datasets, and 46 (36%) trusts did not respond. Clinical directors were asked to estimate the number of specialist hours of direct clinical care scheduled on a typical Sunday and Wednesday in that clinical area. Among responding trusts, moderate agreement was shown between directorate-level questionnaire estimates of specialist hours per ten emergency admissions for both Wednesday and Sunday, and the point prevalence survey data for the same locations (*r* 0·406, p=0·0002 for Wednesday; 0·480, p<0·0001 for Sunday).

Clinical directors were asked how frequently specialists reviewed patients. Daily review of all patients was stated by respondents to be the norm on Sundays for 35 (50%) of 70 acute medical units, 70 (100%) of 70 intensive care units, and 15 (27%) of 55 acute wards; the corresponding values for Wednesdays were 59 (86%) of 69 units, ten (100%) of 70 units, and 32 (58%) of 55 wards, respectively. Consultant vacancies resulting in gaps in cover were reported by 33 (52%) of 63 responding emergency departments, 50 (71%) of 70 acute medical units, 21 (30%) of 70 intensive care units, and 36 (65%) of 55 acute wards.

With 2013–14 Hospital Episodes Statistics data, we fitted the logistic model to in-hospital mortality data from the 141 eligible trusts in England. Results by day of the week are presented in table 2. The estimated weekend effect suggests a 10% increase in mortality for weekend admissions (table 2). This finding is lower than the 11–16% quoted by Freemantle and colleagues,^12^ who included elective admissions in their analysis and used 30 day mortality following admission.

**Figure 3:**
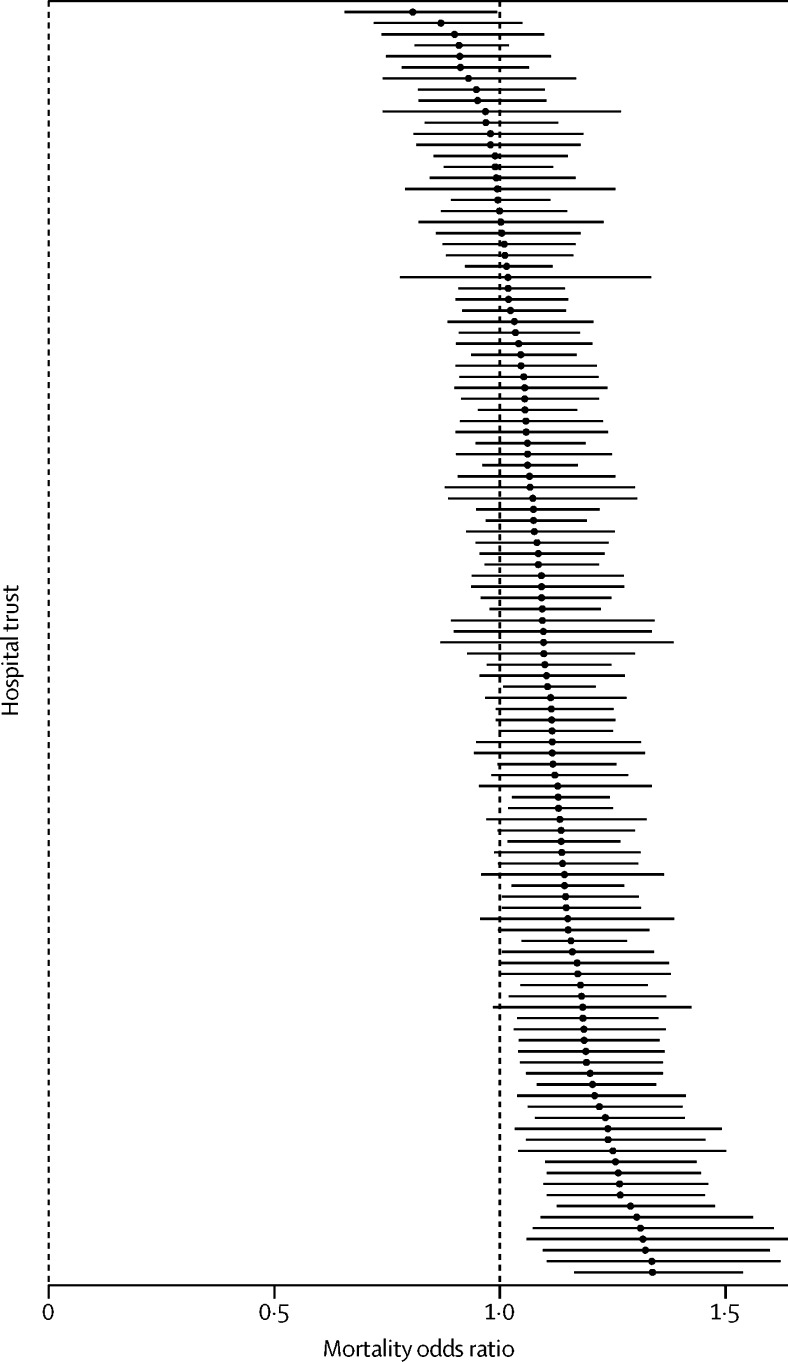
Trust-specific weekend mortality Mortality odds ratios (in increasing order) for weekend to weekday admissions for the 115 trusts contributing to the point prevalence survey. Bars show 95% CIs from logistic regression analysis.

We estimated trust-specific weekend ORs for the 115 trusts contributing to the point prevalence survey. These ratios ranged from 0·81 to 1·34, with 96 (83%) trusts having ORs greater than 1, indicating an estimated excess mortality among weekend admissions (figure 3). There is no systematic association between the weekend mortality effect and trust size (table 1).

**Figure 4:**
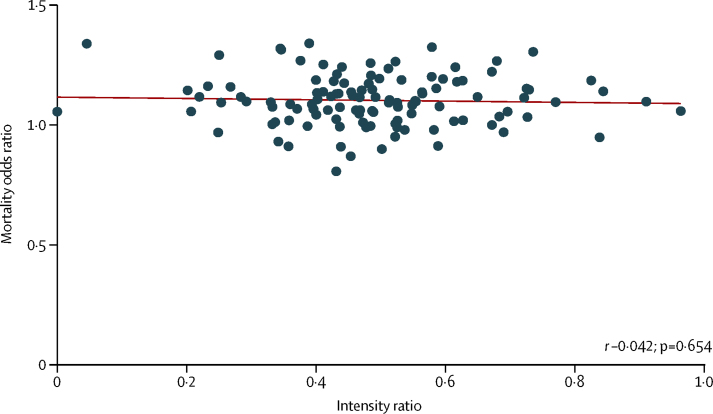
Weekend mortality effects and specialist intensity ratios Mortality odds ratios for weekend to weekday admissions, and specialist intensity ratios for the 115 trusts contributing to the point prevalence survey. Pearson correlations (*r*) and p values are shown.

The correlation between the weekend mortality OR and the Sunday to Wednesday specialist intensity ratio does not show a clear association (figure 4).

## Discussion

To our knowledge, this is the first report of differences between weekend and weekday medical specialist staffing in a health-care system. Our findings show that emergency admissions to English hospitals on a Sunday collectively receive on average less than half the input (hours per ten emergency admissions) from specialists (consultants) of patients admitted on a Wednesday. In terms of numbers of specialists present and attending to patients who had been admitted as emergencies, no hospital achieved even 50% of Wednesday's staffing on a Sunday. Moreover, there was considerable variation between hospitals in specialist input on either a Sunday or a Wednesday, which was not a function of hospital size. The information from the point prevalence survey is supported by that from the directorate-level questionnaire focused on acute specialties: variation in daily ward rounds and gaps in consultant rotas are a source of concern for implementation of 7 day services.

With Wednesday as the reference point, we recorded a 13% excess mortality associated with Sunday admission in the 2013–14 Hospital Episodes Statistics dataset, and 9% on Saturday. However, there was no association between the Sunday to Wednesday specialist intensity ratio and the weekend to weekday mortality ratio. The absence of an association in this preliminary cross-sectional study does not mean that we can discard the hypothesis that the weekend effect is attributable to insufficient presence of specialists, but it does require confirmation from the HiSLAC longitudinal study (phase 2) of concurrent secular changes in intensity and mortality. Caution must be exercised in attributing the weekend effect to a single component in a complex system.^5^

Limitations of our study reflect the methodological challenges of doing health policy research in real time. To improve response rates by replacing surveys with time and motion studies of specialist involvement in the care of acutely ill patients would be prohibitively expensive and carry the risk of the Hawthorne effect, whereas repeated point prevalence surveys would generate respondent fatigue. Hospital Episodes Statistics data do not allow complete casemix adjustment. These limitations have several implications for our results.

Various factors might affect the intensity–mortality association. First, the novel HiSLAC metric of specialist hours per ten emergency admissions might have been degraded by local biases and sampling fluctuations. However, there is little evidence of systematic response-rate bias, and a demonstrable correlation at trust level between the HiSLAC metric and the estimates of intensity offered by individual clinical directors in the directorate-level questionnaire.

Second, several factors suggest that the weekend effect could be attributable to unmeasured casemix differences rather than intensity and quality of care. Notably, although weekend admission is associated with a higher adjusted mortality, the death rate in hospital at the weekend is actually lower than on weekdays.^6^ A single-centre 7 year retrospective analysis incorporating acute physiology has shown that patients admitted at weekends are more severely ill than those admitted on weekdays, explaining most of the surplus mortality.^11^ The weekend effect is also detectable in patients undergoing elective weekend admission^25^, ^26^ and those undergoing elective weekend surgery;^25^, ^27^ elective patients admitted at weekends are likely to differ from routine weekday admissions—eg, those needing more intensive preoperative assessment before surgery scheduled on a Monday.

Third, the impact of specialist care might be modified by variation in other staff levels and support services in hospital (junior doctors, diagnostics, pharmacy, allied health professionals, clerical or administrative services), or in community and social care. The need for a system-wide approach is supported by evidence that hospital-care processes are unreliable at weekends;^16^, ^17^, ^28^, ^29^, ^30^ that more (or more skilled) nurses are associated with better outcomes;^14^, ^20^, ^31^, ^32^ and that in surgical settings, failure to rescue (ie, the inability of the system to respond promptly to patient deterioration) not only explains the difference in outcomes between high and low volume centres,^33^ but also the difference in outcomes from patient safety incidents for patients admitted at weekends compared with weekdays.^34^

Fourth, in view of the paucity of specialist input at weekends across the NHS, there might simply be insufficient variation (inadequate involvement) to detect an effect on patient outcomes in a cross-sectional study. For example, in a hospital endoscopy centre providing 24 h specialist-led care, admission to hospital at a weekend might be associated with more timely treatment than weekdays, not less.^35^ Thus, it might only be possible to detect a beneficial effect from increased specialist intensity at weekends when a sufficient number of hospitals achieve parity of staffing throughout the 7 days, and do this with sufficient staff levels across all days of the week. This notion forms the basis for the second phase of the HiSLAC project, which will examine secular trends in specialist intensity and the weekend effect.

In conclusion, there is a substantial difference between weekend and weekday specialist involvement in the care of patients admitted as emergencies to acute hospitals in England. We are unable to demonstrate an association between specialist staffing and mortality, but would not necessarily expect to do so in the first year of a 5 year longitudinal study. However, this finding suggests the need for caution in attributing the weekend effect mainly to a lack of consultants at weekends. The frequency of reported vacancies in consultant rotas for the acute specialties and the scarcity of acute specialty consultant-led ward rounds at weekends should be addressed in the context of implementation of 7 day services.

## Figures and Tables

**Table 1:** Specialist intensity and weekend mortality, by trust size quintile

	**240–470 beds**	**479–627 beds**	**636–783 beds**	**787–984 beds**	**986–2037 beds**	**All trusts**
**Emergency admissions**						
Number of trusts	23	23	23	23	23	115
Sunday admissions, 2013–14*	43 (38–54)	63 (57–74)	84 (72–97)	94 (80–111)	132 (119,161)	80 (57–106)
Wednesday admissions, 2013–14*	54 (45–64)	81 (67–91)	100 (91,112)	110 (99,132)	162 (144–196)	95 (69–128)
**PPS responses**						
Clinicians surveyed	3168	4210	5807	8572	12 593	34 350
Responders	1480 (47%)	2013 (48%)	2465 (42%)	3857 (45%)	5722 (45%)	15 537 (45%)
Specialist responders	1362 (92%)	1873 (93%)	2304 (93%)	3598 (93%)	5395 (94%)	14 532 (94%)
Exclusions†	118 (8%)	140 (7%)	161 (7%)	259 (7%)	327 (6%)	1005 (6%)
**Specialists attending to emergency admissions on Sunday June 15, 2014**						
Number (% of specialist responders)	157/1362 (12%)	220/1873 (12%)	273/2304 (12%)	430/3598 (12%)	587/5395 (11%)	1667/14 532 (11%)
Hours per specialist present	6·22 (3·46)	5·61 (3·22)	6·18 (3·37)	5·73 (3·45)	5·46 (3·38)	5·74 (3·39)
Specialist intensity†	20·33 (14·59–27·02)	15·07 (9·50–26·99)	22·83 (16·02–29·33)	24·74 (19·15–30·16)	21·49 (15·28–30·18)	21·90 (15·07–29·00)
**Specialists attending to emergency admissions on Wednesday June 18, 2014**						
Number (% of specialist responders)	593/1362 (44%)	855/1873 (46%)	961/2304 (42%)	1549/3598 (43%)	2147/5395 (40%)	6105/14 532 (42%)
Hours per specialist present	4·36 (3·47)	4·00 (3·27)	4·04 (3·22)	3·66 (3·18)	4·06 (3·38)	3·97 (3·31)
Specialist intensity‡	43·85 (34·12–52·46)	37·46 (26·25–56·52)	39·82 (32·04–49·77)	43·75 (34·81–56·95)	46·71 (38·57–71·70)	42·73 (33·37–55·36)
Sunday to Wednesday intensity ratio	0·44 (0·35–0·58)	0·43 (0·40–0·53)	0·55 (0·47–0·67)	0·49 (0·46–0·63)	0·46 (0·36–0·53)	0·48 (0·40–0·58)
Weekend mortality odds ratio	1·09 (1·00–1·25)	1·10 (1·03–1·14)	1·11 (1·05–1·19)	1·11 (1·01–1·20)	1·10 (1·05–1·13)	1·10 (1·03–1·17)

Data are median (IQR), n (%), n/N (%), or mean (SD) unless otherwise specified. PPS=point prevalence survey.

* For each trust, emergency admissions are the mean number of Sunday or Wednesday emergency admissions over 2013–14. The median (IQR) refers to variation in these averages across different trusts.

† Includes respondents without certificates of completion of specialist training, and two incomplete responses.

‡ Total specialist hours per ten emergency admissions, corrected for response rate.

**Table 2:** Relative odds of in-hospital death by day of admission, adjusted for casemix

	**Odds ratio (95% CI)**	**p value**
Monday	1·02 (1·01–1·04)	0·013
Tuesday	1·00 (0·98–1·02)	0·852
Wednesday (reference)	1	··
Thursday	1·02 (1·00–1·04)	0·032
Friday	1·01 (0·99–1·03)	0·279
Saturday	1·09 (1·07–1·12)	<0·0001
Sunday	1·13 (1·10–1·15)	<0·0001
Weekend effect*	1·10 (1·08–1·11)	<0·0001

* Obtained from the admission day odds ratios, as described in the text.
