# Supplementary material for: Weekend specialist intensity and admission mortality in acute hospital trusts in England: a cross-sectional study
Source: Lancet. 2016 Jul 9;388(10040):178–86. doi: 10.1016/S0140-6736(16)30442-1 (PMC4945602; doi:10.1016/S0140-6736(16)30442-1)

THE LANCET

Supplementary appendix

This appendix formed part of the original submission and has been peer reviewed. We post it as supplied by the authors.

Supplement to: Aldridge C, Bion J, Boyal A, et al. Weekend specialist intensity and admission mortality in acute hospital trusts in England: a cross-sectional study. *Lancet* 2016; published online May 10. http://dx.doi.org/10.1016/S0140-6736(16)30442-1.

HiSLAC Phase 1 Appendix

Appendix 1 The HiSLAC Collaborative

Project Management Committee: Julian Bion (Chief Investigator), Cassie Aldridge, Peter Rees, Chris Roseveare, Tim Evans, Mark Temple, Mike Clancy, Richard Lilford, Joanne Lord, Sam Watson, Alan Girling, Gavin Rudge, Jianxia Sun, Amunpreet Boyal, Russell Mannion, Carolyn Tarrant, Liz Sutton, Janet Willars, Sarah Chew, David Kocman, Elisa Liberati, Emma Angell, Yen-Fu Chen, Louise Rowan, Felicity Evison, Simon Baldwin, Stephen Perks.

Oversight and Governance Committee: Michael Rawlins (Chair), Jennifer Dixon (Health Foundation), Peter Lees (Faculty of Medical Leadership and Management), Paddy Storrie (Patient representative), Alastair Henderson (Academy of Medical Royal Colleges), Matt Sutton (health economics), Naomi Fulop (healthcare organisation and management).

Scientific Advisory Board: Mary Dixon-Woods, Derek Bell, Andrew Goddard, Mike Grocott, Kathy Rowan, Julie Moore, Deborah Williams, Simon Bennett, Keith Willett, Mike Durkin, Jerry Nolan, David & Kay Schofield, Tim Doran, Carl Macrae.

Local Project Leads: John Hollingsworth and Linda Matthew (Aintree University Hospital NHS Foundation Trust). Cornelle Parker (Airedale NHS Foundation Trust). Pasha Nisar (Ashford and St Peter's Hospitals NHS Foundation Trust). Magda Smith and Stephen Burgess (Barking, Havering and Redbridge University Hospitals NHS Trust). Marcus Pittman (Basildon and Thurrock University Hospitals NHS Foundation Trust). Shwe Zin Chit Pan (Bedford Hospital NHS Trust) Mark O'Donnell (Blackpool Teaching Hospitals NHS Trust). Natalie Walker and Leonidas Zachariades (Bolton NHS Foundation Trust). Wendy Munro (Brighton and Sussex University Hospitals NHS Trust). Andrew Huang (Buckinghamshire Healthcare NHS Trust). Andrew Willis (Burton Hospitals NHS Foundation Trust). Rehan Naseer (Calderdale and Huddersfield NHS Foundation Trust). Chris Adcock (Cambridge University Hospitals NHS Foundation Trust). Bronwyn Kerr (Central Manchester University Hospitals NHS Foundation Trust). Allan Harkness (Colchester Hospital University NHS Foundation Trust). Frank Joseph (Countess Of Chester Hospital NHS Foundation Trust). Martyn Farrer (City Hospitals Sunderland NHS Foundation Trust). Michael Jones (County Durham and Darlington NHS Foundation Trust). Nnenna Osuji (Croydon Health Services NHS Trust). Aref Rastegar and Lanitha Srikugan (Dartford and Gravesham NHS Trust). Richard Donnelly (Derby Hospitals NHS Foundation Trust). Timothy Noble (Doncaster and Bassetlaw Hospitals NHS Foundation Trust). Richard Jee (Dorset County Hospital NHS Foundation Trust). Kevin Baynes (Ealing Hospital NHS Trust). Neil Harris and Jane McCue (East and North Hertfordshire NHS Trust). Sanjeev Srivastava (East Cheshire NHS Trust). Paul Stevens (East Kent Hospitals University NHS Foundation Trust). Charles Thomson (East Lancashire Hospitals NHS Trust) James Wilkinson and Harry Walmsley (East Sussex Healthcare NHS Trust). Guan Lim (Epsom and St Helier University Hospitals NHS Trust). Christopher Scott (Gateshead Health NHS Foundation Trust) Gordon Wood (George Eliot Hospital NHS Trust). Debesh Mukherjee (Great Western Hospitals NHS Foundation Trust). Richard Leach and David M Wood (Guy's and St Thomas' NHS Foundation Trust and King's Health Partners). Ben Cresswell (Hampshire Hospitals NHS Foundation Trust). Peter Hammond (Harrogate and District NHS Foundation Trust). Rifat Rashid and Matthew Cooke (Heart Of England NHS Foundation Trust). Tiina Tamm (Heatherwood and Wexham Park Hospitals NHS Foundation Trust). Anitha Mathews (Hinchingbrooke Health Care NHS Trust). Emma Rowland (Homerton University Hospital NHS Foundation Trust). William Oldfield (Imperial College Healthcare NHS Trust). Victor Lawrence (Isle Of Wight NHS Trust). Raja Reddy (Kettering General Hospital NHS Foundation Trust). William Bernal (King's College Hospital NHS Foundation Trust). Jane Wilson (Kingston Hospital NHS Trust). Graham Johnson (Leeds Teaching Hospitals NHS Trust). David Sulch (Lewisham and Greenwich NHS Trust). Peter Albert (Luton and Dunstable Hospital NHS Foundation Trust). Vinay Bhatia and Clive Lawson (Maidstone and Tunbridge Wells NHS Trust). Graeme Sanders (Medway NHS Foundation Trust). Shirley Hammersley (Mid Cheshire Hospitals NHS Foundation Trust). Jayachandran Radhakrishnan, Aung Lwin and Kevin Kiff (Mid Essex Hospital Services NHS Trust). Chipo Ndlovu (Mid Staffordshire NHS Foundation Trust). Ian Carr and Sathish Bhasker (Mid Yorkshire Hospitals NHS Trust). Ijaz Mehdi (Milton Keynes Hospital NHS Foundation Trust). Neil Burgess (Norfolk and Norwich University Hospitals NHS Foundation Trust). Kiaran Flanagan (North Bristol NHS Trust). Denis Burke (North Cumbria University Hospitals NHS Trust). Nick Roper (North Tees and Hartlepool NHS Foundation Trust). Amanda Bisset (Northampton General Hospital NHS Trust). Roope Manhas (Northern Devon Healthcare NHS Trust). David Evans (Northumbria Healthcare NHS Foundation Trust). Keith Girling (Nottingham University Hospitals NHS Trust). James Price (Oxford University Hospitals NHS Trust). Nick Tierney (Pennine Acute Hospitals NHS Trust). Callum Gardner (Peterborough and Stamford Hospitals NHS Foundation Trust). Sam

Waddy and Emma Rayment (Plymouth Hospitals NHS Trust). Phil Young (Portsmouth Hospitals NHS Trust). William Orr (Royal Berkshire NHS Foundation Trust). David Friedericksen (Royal Cornwall Hospitals NHS Trust). James Hobbs and Martin Cooper (Royal Devon and Exeter NHS Foundation Trust). Rupert Negus (Royal Free London NHS Foundation Trust). Kathryn Clark (Royal Liverpool and Broadgreen University Hospitals NHS Trust). Michelle Gallagher (Royal Surrey County Hospital NHS Foundation Trust). Richard Warner (Salford Royal NHS Foundation Trust). Stuart Henderson (Salisbury NHS Foundation Trust). Matthew Lewis (Sandwell and West Birmingham Hospitals NHS Trust). Andrew Gibson (Sheffield Teaching Hospitals NHS Foundation Trust). Joanna Richardson (Sherwood Forest Hospitals NHS Foundation Trust). Richard Heinink (Shrewsbury and Telford Hospital NHS Trust). David Sinclair (South Devon Healthcare NHS Foundation Trust). Sath Nag (South Tees Hospitals NHS Foundation Trust). Jyothi Nippani (South Warwickshire NHS Foundation Trust). John Kinnear (Southend University Hospital NHS Foundation Trust). Graham Butcher (Southport and Ormskirk Hospital NHS Trust). Jane Evans (St George's Healthcare NHS Trust). Peter Williams (St Helens and Knowsley Hospitals NHS Trust). James Catania, Sengottiyan Chandrasekaran and Richard Ellis (Stockport NHS Foundation Trust). Martin Pattrick (Tameside Hospital NHS Foundation Trust). Matthew Hayman (Taunton and Somerset NHS Foundation Trust). Hassan Paraiso (The Dudley Group NHS Foundation Trust). Mark Edwards (The Hillingdon Hospitals NHS Foundation Trust). Yvonne Barlow (The Princess Alexandra Hospital NHS Trust). Mark Blunt (The Queen Elizabeth Hospital, King's Lynn NHS Foundation Trust). David Harling (The Rotherham NHS Foundation Trust). Earl Williams (The Royal Bournemouth and Christchurch Hospitals NHS Foundation Trust). Lee Dowson (The Royal Wolverhampton NHS Trust). Clarissa Murdoch (The Whittington Hospital NHS Trust). Sanjiv Jain (United Lincolnshire Hospitals NHS Trust). Nicoletta Gaston (University College London Hospitals NHS Foundation Trust). Michael Berry (University Hospitals Birmingham NHS Foundation Trust). Nick Coleman (University Hospital Of North Midlands NHS Trust). Tim Cooksley (University Hospital Of South Manchester NHS Foundation Trust). Mike Clancy and Chris Roseveare (University Hospital Southampton NHS Foundation Trust). Rebecca Thorpe and Emma Redfern (University Hospitals Bristol NHS Foundation Trust). Mike Iredale (University Hospitals Coventry and Warwickshire NHS Trust). Mark Ardron (University Hospitals Of Leicester NHS Trust). Andrew Higham (University Hospitals Of Morecambe Bay NHS Foundation Trust). Michael Van Der Watt (West Hertfordshire Hospitals NHS Trust). Helen Burgess and Emma Rowlandson (West Middlesex University Hospital NHS Trust). Paul Molyneux (West Suffolk NHS Foundation Trust). Robert Haigh (Western Sussex Hospitals NHS Trust). Melanie Maxwell (Wirral University Teaching Hospital NHS Foundation Trust). Steve Graystone (Worcestershire Acute Hospitals NHS Trust). Rupert Ransford (Wye Valley NHS Trust).

Appendix 2. High-intensity Specialist Led Acute Care (HiSLAC) study Point Prevalence Survey questions and explanatory text. Survey was launched Thursday June 19th 2014 and closed at 23:59 Monday July 14th.

**Q1**	**Are you a consultant or a Staff or Associate Specialist doctor with a CCT (or equivalent)?**
**A1**	Yes/No If a no answer was given respondents were directed to Q4
**Note1**	*CCT or equivalent (for example CESR) means individuals registered on the General Medical Council's specialist register. Locum consultants without a CCT and not on the specialist register should answer 'no' to this question.*
**Q2.1**	**Last Sunday 15th June were you physically present in the hospital at any point between 08:00h-20:00hrs providing direct clinical care to patients who had been admitted for their current admission episode as an emergency to any in-patient bed including CDUs/Observation wards?**
**A2.1**	Yes/No If a no answer was given respondents were directed to Q3.1
**Note2.1**	*The focus of this survey is patients admitted to an in-patient bed, which includes CDUs and observation wards. The care given to patients in the ED before admission is not part of this survey.*
**Q2.2**	**On that Sunday, approximately how many hours did you spend providing direct clinical care to emergency admissions?**
**A2.2**	Select: 1/2/3/4/5/6/7/8/9/10/11/12 or more
**Note2.2**	*We are only interested in the time dedicated to emergency admissions (even though your contracted PAs may include a commitment to routine ward care and review). Thus if half the patients you reviewed were elective admissions, please do not include them in your assessment of time spent, even though you were in the hospital during the survey period. For example, specialists working in AMUs or ICUs will generally spend all their time on emergency admissions; surgeons, anaesthetists or radiologists might have more elective admission patients and fewer emergencies.*<br>*Please round up your response to the nearest hour*
**Q2.3**	**Were you the specialist responsible for continuing care of these patients? (ie: 'named consultant')**
**A2.3**	Select: Yes/No/Some
**Note2.3**	*The purpose of this question is to determine the extent to which you may be familiar with the patients' problems.*
**Q2.4**	**Which of the following locations best describe where you spent most time delivering direct patient care?**
**A2.4**	Selected from the following, no restrictions were given on the number that could be selected: Acute Medical Unit / Acute Surgical Unit / Adult Medical Ward / Adult Surgical Ward / Combined Med/Surg Acute Unit / ED Clinical Decision Unit / Endoscopy Unit / High Dependency Unit / Intensive Care Unit / Laboratories / Obstetrics or Gynaecology Ward / Operating Theatre / Paediatrics Wards/units / Radiology / Specialist Wards or Units / Other
**Note2.4**	*Only list one location if that is where you spent most of your time. We have not included locations which only provide elective or outpatient care.*
**Q3.1**	**Last Wednesday 18th June were you physically present in the hospital at any point between 08:00h-20:00hrs providing direct clinical care to patients who had been admitted for their current admission episode as an emergency to any in-patient bed including CDUs/Observation wards?**
**A3.1**	Yes/No If a no answer was given respondents were directed to Q4
**Note3.1**	*The focus of this survey is patients admitted to an in-patient bed, which includes CDUs and observation wards. The care given to patients in the ED before admission is not part of this survey*
**Q3.2**	**On that Wednesday, approximately how many hours did you spend providing direct clinical care to emergency admissions?**
**A3.2**	Select: 1/2/3/4/5/6/7/8/9/10/11/12 or more
**Note3.2**	*We are only interested in the time dedicated to emergency admissions (even though your contracted PAs may include a commitment to routine ward care and review). Thus if half the patients you reviewed were elective admissions, please do not include them in your assessment of time spent, even though you were in the hospital during the survey period. For example, specialists working in AMUs or ICUs will generally spend all their time on emergency admissions; surgeons, anaesthetists or radiologists might have more elective admission patients and fewer emergencies.*<br>*Please round up your response to the nearest hour*
**Q3.3**	**Were you the specialist responsible for continuing care of these patients? (ie: 'named consultant')**
**A3.3**	Select: Yes/No/Some
**Note3.3**	*The purpose of this question is to determine the extent to which you may be familiar with the patients' problems.*
**Q3.4**	**Which of the following locations best describe where you spent most time delivering direct patient care?**
**A3.4**	Selected from the following, no restriction were given on the number that could be selected: Acute Medical Unit / Acute Surgical Unit / Adult Medical Ward / Adult Surgical Ward / Combined Med/Surg Acute Unit / ED Clinical Decision Unit / Endoscopy Unit / High Dependency Unit / Intensive Care Unit / Laboratories / Obstetrics or Gynaecology Ward / Operating Theatre / Paediatrics Wards/units / Radiology / Specialist Wards or Units / Other
**Note3.4**	*Only list one location if that is where you spent most of your time. We have not included locations which only provide elective or outpatient care.*
**Q4**	**Please identify your main speciality (only 1 choice)**<br>**Please answer this question even if you had no clinical duties on the two days identified previously.**
**A4**	Selected from one of the following: Acute Internal Medicine / Allergy / Anaesthetics / Cardio-thoracic Surgery / Cardiology / Chemical Pathology / Clinical Genetics / Clinical Neurophysiology / Clinical Pharmacology and Therapeutics / Dermatology / Emergency Medicine / Endocrinology and Diabetes / Gastroenterology / General (Internal) Medicine / General Surgery / Genito-urinary Medicine / Geriatric Medicine / Haematology / Histopathology / Immunology / Infectious Diseases / Intensive

	Care Medicine / Medical Microbiology / Neurology / Neurosurgery / Nuclear Medicine / Obstetrics & Gynaecology / Oncology / Ophthalmology / Oral and maxillo-facial Surgery / Otolaryngology / Paediatrics / Palliative Medicine / Pharmaceutical Medicine / Plastic Surgery / Radiology / Rehabilitation Medicine / Renal Medicine / Respiratory Medicine / Rheumatology / Sport and Exercise Medicine / Trauma and Orthopaedic Surgery / Tropical Medicine / Urology / Other
**Note4**	*We realise that many specialists hold more than one CCT, whereas others may practice full time in a speciality which at the date of completion of training did not provide a CCT (eg: acute medicine, intensive care medicine). Please choose that speciality which best reflects your practice on the two survey days.*

Appendix 3. High-intensity Specialist Led Acute Care (HiSLAC) study Directorate Level Questionnaire

The questionnaire was launched on the 7th October 2014. The last data collected was 14th January 2015.

	Please note that 'Specialist' = consultants + associate specialists (CCT holder or article 14 CESR). It does not include Trust doctors without a CCT, or trainees. DCC = direct clinical care	**Emergency Department + CDU (A)**	**Acute Medical Unit (B)**	**Intensive Care Units (C)**	**Acute medical Wards (D)**
**On a weekday**					
**Q040**	**How many specialists are normally physically present in this clinical area providing direct clinical care on a weekday during the daytime (08:00-20:00hrs)? (Please include only those with contractual DCC-PAs for this area, not visiting specialists).**				
**Q140**	**On average, how many hours would a specialist normally spend providing direct clinical care in this area, on a typical weekday during the daytime (08:00-20:00h)?**				
**Q070**	**Do specialists provide direct clinical care on single days (e.g. 'physician of the day' model) or in blocks of 2 days, or 3 or more days? Answer 1, 2 or 3**<br>**1)** Single days<br>**2)** Blocks of two days<br>**3)** Blocks of three or more days				
**Q120**	**How often are patients in this clinical area reviewed by a specialist on weekdays? Answer 1, 2 or 3**<br>**1)** All patients are reviewed at least once each day by a specialist<br>**2)** Selected patients are reviewed at least once each day by a specialist<br>**3)** Patients are reviewed by a specialist less frequently than daily				
**On a Sunday**					
**Q050**	**How many specialists are normally present in this clinical area providing direct clinical care on a Sunday during the daytime (08:00-20:00hrs)? (Please include only those with contractual DCC-PAs for this area, not visiting specialists).**				
**Q150**	**On average, how many hours would a specialist normally spend providing direct clinical care in this area, on a Sunday during the daytime (08:00-20:00h)?**				
**Q080**	**Do specialists provide direct clinical care on Sundays only (e.g. 'physician of the day' model) or in blocks of 2 days, or 3 or more days incorporating Sunday? Answer 1, 2 or 3**<br>1) Single Sunday<br>2) Blocks of two days (eg whole weekend)<br>3) Blocks of three or more days incorporating Sunday				
**Q110**	**How often are patients in this clinical area reviewed by a specialist on Sundays? Answer 1, 2 or 3**<br>1) All patients are reviewed at least once each Sunday by a specialist<br>2) Selected patients are reviewed at least once on Sunday by a specialist<br>3) Patients are reviewed by a specialist less frequently than daily				
**Q060**	**What model of specialist care is provided in this clinical area on Sundays? Answer 1, 2 or 3**<br>1) Only specialists with weekday daytime sessions in this clinical area provide care on Sundays<br>2) Specialists whose weekday daytime sessions are exclusively in a different clinical area provide care in this area on Sundays<br>3) Care on Sunday is provided by specialists both with and without weekday sessions in this area				
**Q090**	**Please indicate the type of specialist weekend rota: Answer 1, 2 or 3**<br>1) On call<br>2) Shift work (ie scheduled to be actually present in the clinical area providing direct clinical care for a period)<br>3) Combination of both				
**Q092**	**What is the usual Sunday working frequency for specialists with contracted DCCs for this clinical area? [eg 1:4, 1:6 etc]**				
**Weekday or Sunday**					
**Q100**	**Do you currently have consultant vacancies resulting in gaps in the specialist rota in this area?** Yes/No				
**Q130**	**Please enter any additional general comments if you wish:**				

Appendix 4: Geographical distribution of participating and non-participating Trusts

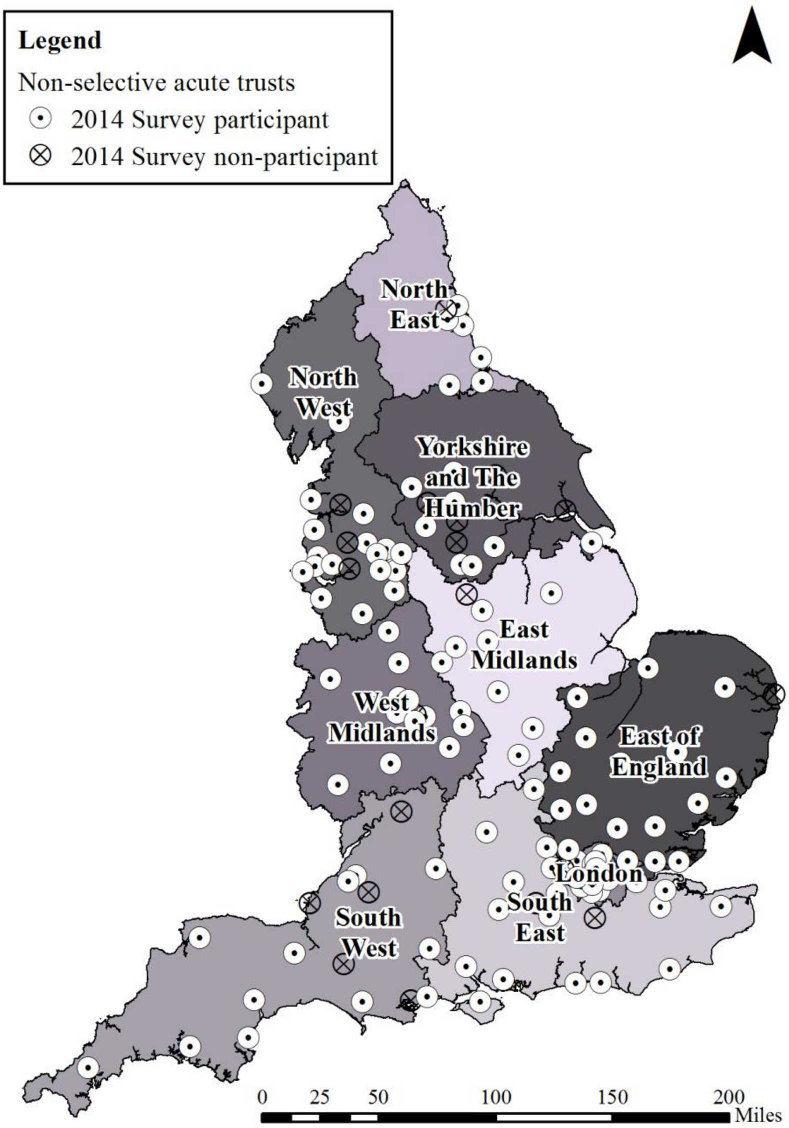

Supplement: Supplementary appendix [file mmc1.pdf]
